# COVID-19 Toes and Other Skin Lesions During the Pandemic: Emerging Entities?

**DOI:** 10.1177/15347346211011843

**Published:** 2022-12

**Authors:** Prashanth R. J. Vas, George S. Georgiadis, Nikolaos Papanas

**Affiliations:** 1 King’s College NHS Foundation Trust, London, UK; 2 King’s Health Partners’ Institute of Diabetes, Endocrinology and Obesity, London, UK; 3 Department of Vascular Surgery, Democritus University of Thrace, University Hospital of Alexandroupolis, Thrace, Greece; 4 Diabetes Centre-Diabetic Foot Clinic, Democritus University of Thrace, Alexandroupolis, Greece

**Keywords:** COVID-19, coronavirus, cutaneous, perniosis

## Abstract

There is accumulating evidence to indicate an association between coronavirus infectious disease 2019 (COVID-19) and clusters of incident cutaneous eruptions. Of these, chilblains-like perniosis have received widespread medical and media attention. These typically affect the toes, and have been called “COVID-toes.” Other acral lesions such as large bullae have also been reported. However, a definitive causal relationship with the severe acute respiratory syndrome coronavirus 2 has not yet been definitively proven, nor has a pathogenic mechanism been established. These episodes are self-limiting, but we need to know whether long-term sequelae exist.

## Editorial

The coronavirus infectious disease 2019 (COVID-19) pandemic caused by the severe acute respiratory syndrome coronavirus 2 (SARS-CoV-2) is predominantly a respiratory tract infection characterized by high infection rate, pulmonary infiltrates, and high mortality among older and immunocompromised individuals.^1^ Although the lungs and the immune system are primarily affected, it is now apparent that the virus may also affect other systems, frequently involving the cardiovascular, gastrointestinal tract, and nervous systems.^2^ Inflammation in these is linked with circulatory impairments in the local microvasculature, manifesting as thrombosis in the pulmonary circulation,^3^ and other parts of the vascular tree.^4,5^ These are accompanied by hypercoagulability^6^ along with increased blood neutrophils and neutrophil extracellular traps.^7^ On histopathological examination, endothelial inflammation and diffuse microvascular necrosis have been noted.^2,3,8^

Importantly, there are also cutaneous manifestations of COVID-19, first described in an Italian cohort of hospitalized patients. These appear to include maculopapular, urticarial, and vesicular rashes, which may be generalized.^9^ However, the most reported cutaneous feature is the chilblain-like perniosis in the distal parts of the lower extremities, particularly in the feet, the so-called “COVID-toes.”^10-12^ Such perniosis was first reported in children and young individuals, but it may be encountered in all ages.^10,11^ Medical reports of the so-called “clusters of pernio” have received significant attention and wide publicity.^12^ Of interest, most individuals with COVID-toes only had mild respiratory symptoms, frequently presenting only with these cutaneous lesions.^9,11^

Zinder et al^13^ have reported 3 cases of lower extremity bullae in patients hospitalized with severe COVID-19. All patients had serious comorbidities, including diabetes mellitus, obesity, renal dysfunction, cardiac failure, and others.^13^ The bullae resolved with supportive wound care and did not impact their overall clinical course and outcomes.

Importantly, the skin not only provides protection to tissues underneath, but it is also the largest microvascular bed. Cutaneous structures (endothelium, small vessel smooth muscles, and sebaceous glands) express the angiotensin-converting enzyme-2 (ACE-2) receptor, used by the virus for cell entry.^14^ Hence, it is plausible that cutaneous lesions are a direct manifestation of virus-induced microvascular inflammation. However, in many studies, the association with SARS-CoV-2 has been circumstantial or uncertain, perhaps partly attributable to diagnostic shortcomings of viral diagnostic assays.^9^ Certainly, the absence of cold exposure points to a diagnosis other than classical perniosis, encouraging the investigation of new causes.^15^

In this context, several questions need to be answered. First, we need to provide a definitive answer on the probable causality. Second, we need to know whether similar or other skin lesions may occur during future waves of the pandemic. Moreover, an inquiry into patient characteristics and comorbidities predisposing to skin lesions is desirable. At present, such perniosis appears to be self-limiting with spontaneous recovery and without any short-term sequelae, but medium- and long-term outcomes are yet to be determined. Additionally, we should decipher if COVID-toes are associated with lung inflammation and/or fibrosis, as well as with systemic endothelial injury, as in perniosis associated with connective tissue disorders.^16^

Finally, improved insights into the underlying mechanisms are warranted. There is histopathological evidence of direct microvascular inflammation analogous to pulmonary lesions,^8,17^ but this has not been consistent.^11^ Indeed, preexisting endothelial dysfunction may complicate the clinical course and contribute to disproportionate hypoxia in older individuals and those with diabetes and obesity.^18,19^ Still, it is unclear whether such perturbations underlie perniosis. Definitely, they cannot explain the higher frequency of COVID-toes in young adults. As dedicated to the science of wound healing,^20,21^ this journal will seek to contribute to further knowledge.

In conclusion, emerging evidence indicates an association between COVID-19 and clusters of incident cutaneous eruptions, especially perniosis. These most commonly affect the toes. However, the causal relationship has not yet been definitively proven. Indeed, we need to know more about the accuracy of viral tests and the role of patient comorbidities. Meanwhile, however, such lesions should trigger immediate self-isolation and urgent investigation for COVID-19, even in the absence of classical symptoms.
